# Understanding the impact of *Klebsiella pneumoniae* K-Antigen based MAPS vaccine design on the immune response in animal models

**DOI:** 10.1371/journal.ppat.1014289

**Published:** 2026-06-10

**Authors:** Elena Palmieri, Gianina Florentina Belciug, Francesca Nonne, Luisa Massai, Silvia Valensin, Antonella De Rosa, Francesco Berlanda Scorza, Simona Rondini, Martina Carducci, Omar Rossi, Francesca Micoli, Carlo Giannelli

**Affiliations:** 1 GSK Vaccines Institute for Global Health, Siena, Italy; 2 Toscana Life Sciences, Siena, Italy; Harvard Medical School, UNITED STATES OF AMERICA

## Abstract

MAPS technology represents an innovative approach for the development of polysaccharide-based vaccines, relying on the affinity interaction between biotin and rhizavidin (rhavi), with potential for enhanced coverage through the incorporation of pathogen-specific proteins. Due to its flexibility, this platform is particularly attractive for the development of multivalent vaccines, as it is required for *Klebsiella pneumoniae*, a multidrug-resistant bacterium highlighted as prevalent cause of neonatal sepsis in low- and middle-income countries, against which no vaccines are currently available. In this work MrkA, a potential protective pathogen-specific protein antigen, was combined in a MAPS complex with K2 as model for *Klebsiella* K-antigen. The impact of sugar chain length, protein to polysaccharide ratio, and MAPS complex size on the immune response elicited in animal models was evaluated. Results showed that longer polysaccharides and higher protein/K-antigen ratios enhanced the immunogenicity in rabbits. Different proteins, i.e., rhavi alone and CP1-rhavi from *Streptococcus pneumoniae*, were also evaluated as carrier, showing that the protein antigen fused to rhavi can play a significant role on the resulting MAPS polysaccharide specific immunogenicity. Passive transfer of rabbit polyclonal sera from the most immunogenic MAPS complex was able to protect mice from challenge with a K2 clinical isolate. This work supports the rational design of a K-antigen MAPS-based vaccine against *Klebsiella pneumoniae*.

## Author summary

In this study we investigated a flexible vaccine approach that combines bacterial surface sugars to proteins to enhance immune protection. *Klebsiella pneumoniae* is a growing cause of severe, drug-resistant infections, particularly in newborns in low- and middle-income countries, and no licensed vaccine currently exists. By testing how sugar chain length, the proportion of protein, and resulting construct size affected immune responses in animals, we identified design features that improve antibody generation: longer sugar chains and higher protein-to-sugar ratios increased responses in rabbits. We also showed that fusing a pathogen-specific protein to the sugar scaffold can further enhance ability of the vaccine to protect. Antibodies produced by the best formulations protected mice from infection with a clinical *Klebsiella* strain when transferred passively. Placed in the broader context, these results offer a practical strategy for rationally designing multivalent vaccines that combine surface sugars and proteins to target difficult bacterial threats. The findings could guide development of vaccines against *Klebsiella* and other multidrug-resistant bacteria.

## Introduction

*Klebsiella pneumoniae* (Kp) is the primary causative agent of neonatal sepsis worldwide [1,2]. In low- and middle-income countries (LMICs), where the majority of cases are caused by hospital-associated drug-resistant classical Kp strains [3], the rates of neonatal septicemia are 3–20 times higher than in industrialized countries [4]. Kp has been also highlighted as the second leading cause of antimicrobial resistance (AMR)-related deaths globally, and the leading cause in sub-Saharan Africa [5]. Majority of neonatal deaths occur between 72 hours and 28 days of life [6], thus a maternal vaccine could play a decisive role both preventing the infection and in reducing antimicrobial use. It has been shown that a hypothetical Kp maternal vaccine with 70% efficacy could reduce neonatal sepsis deaths in many LMICs by ~ 15% [7]. Currently, no vaccines against Kp are available yet, but several vaccines are under preclinical development, with a few moved to clinical phase [8–10].

Capsular polysaccharides, known as K-antigens (KAg), constitute potential antigens for vaccine design, with a number of studies demonstrating ability of anti-KAg antibodies to confer protection against Kp in animal models of infection [8,9]. KAg, present on Kp surface, are known to promote immune evasion, enhancing bacterial resistance to intracellular killing [11,12]. To date, nearly 80 types of capsules, which differ from one another by the structure and components of the repeating polysaccharide unit [13], have been identified and 186 K-loci from Kp genomes have been proposed based on unique gene sequences in the biosynthesis locus [14–16]. This diversity calls for a multi-valent vaccine and, according to recent estimates, a 20-valent KAg-based vaccine could theoretically cover ≥70% of infections in all target regions [17].

MAPS platform represents an innovative technology for development of polysaccharide-based high-valency vaccines. This platform has already been proposed for many multi-valent vaccines, e.g., against *Salmonella* [18,19], *Shigella* [20], Group B *Streptococcus* (GBS) [21]*,* and a 24-valent pneumococcal vaccine is currently in clinical development [22–24]. MAPS technology is based on the site-specific and high-affinity noncovalent linkage between biotin moieties, introduced on the polysaccharide backbone, and the biotin-binding rhizavidin (rhavi) moiety genetically fused to a protein of interest [25]. The use of a pathogen specific protein represents an advantage to possibly increase vaccine coverage and reduce complexity. This approach can be easily extended to different polysaccharide-protein systems allowing rapid development of multi-valent vaccines.

In this perspective, MrkA, the major component of Kp type 3 fimbriae, can be proposed in combination with Kp KAg through MAPS technology. MrkA is surface exposed protein well conserved among different serotypes, and its ability to protect mice from Kp lethal challenge has been reported [26]. Recently a rhavi form of MrkA fused to the D2 segment of *Pseudomonas aeruginosa* flagellin B FlaBD2 [27] (here named as Rhavi-FlaBD2-MrkA), has been designed and proposed for an O-antigen-based MAPS combination vaccine against both Kp and *Pseudomonas aeruginosa* [28]. Rhavi is an avidin-like protein that forms non-covalent dimers [29], and in this particular design is fused to the N-terminus of FlaBD2-MrkA, maintaining its ability to adopt a dimeric quaternary structure [28].

Here, we have developed a KAg-Rhavi-FlaBD2-MrkA MAPS, selecting K2 as model KAg. K2 serotype is often found in classical and hypervirulent Kp strains and is identified among the most prevalent serotypes responsible for neonatal sepsis in LMICs [17,30,31]. In this work we aimed to identify which structural features can impact the response elicited by a KAg MAPS vaccine. Within this scope, K2-Rhavi-FlaBD2-MrkA MAPS complexes with different characteristics (KAg length, protein to KAg ratio and MAPS complex size) were designed, fully analytically characterized and tested in multiple animal models. Results from this work will be extended to additional KAg, helping to rationally design a multi-valent MAPS based vaccine against Kp.

## Results

### Testing different chemistries for KAg biotinylation

Two different chemistries were tested for K2 biotinylation: activation of hydroxyl groups along the polysaccharide chain through a cyanylation reaction with 1-cyano-4-dimethylaminopyridinium tetrafluoroborate (CDAP) or activation of carboxylic groups along the polysaccharide chain with 1-Ethyl-3 (3dimethylaminopropyl)carbodiimide/Sulpho N-Hydroxysuccinimide (EDAC/S-NHS) before addition of Amine-PEG3-Biotin linker.

In both cases, reaction conditions were controlled to have an activation degree close to 1% not to impact K2 epitopes. K2_CDAP_-biotin resulted to contain 17.0 nmol biotin/mg of K2, corresponding to 1.1% of K2 repeating unit (RU) biotinylated, while K2_EDAC_-biotin had 13.7 nmol/mg K2, corresponding to 0.9% of K2 RU derivatized.

In order to check intermediates stability, both samples were incubated at 4, 25 and 40 °C for 21 days. At selected time points (3, 6, 9, 13, 17 and 21 days), both saccharide and biotin contents were quantified to verify the stability of the isourea or amide bond, following the eventual loss of biotin.

**Fig 1:**
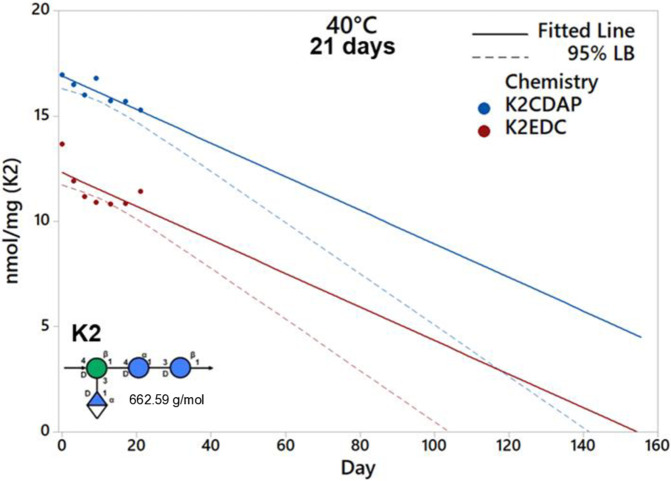
Stability of biotinylated K2 post CDAP or EDAC activation. At 40 °C, a reduction of biotin content is observed overtime with not significantly different rate for the two chemistries used (LB = Lower Bound). ANCOVA analysis is reported in S2 Fig.

Only at 40 °C we could identify a significant decreasing trend of RU biotinylation level for both chemistries used (Fig 1), while at 4 and 25 °C both intermediates resulted stable, therefore a comprehensive kinetic model [32] could not be found (S1 Fig). Using Analysis of Covariance (ANCOVA), it was verified that instability was independent from the type of chemistry used for polysaccharide biotinylation (the slopes of the two trends were not significantly different).

Based on these results, CDAP chemistry with 1,4-diazabicyclo[2.2.2]octane (DABCO) buffering system [33] was selected for the subsequent MAPS complexes generation, because of its simpler process compared to EDAC/NHS chemistry (one step, no pH adjustment), making the fine-tuning of reaction conditions easier and reducing process variability.

### K2 MAPS complex dose-ranging studies in different animal species, with and without Alhydrogel

A K2-Rhavi-FlaBD2-MrkA MAPS complex (S3 Fig) was synthesized with native K2 (530 kDa) activated with biotin (1.2% of polysaccharide RU), resulting in a protein/polysaccharide w/w ratio of 3.0 (corresponding to a molar ratio of protein/polysaccharide RU of 0.017). K2-Rhavi-FlaBD2-MrkA MAPS complex was tested at 5 different K2 doses (5-fold dilutions in the range 2.4-1500 ng) with and without Alhydrogel in mice and rabbits.

**Fig 2:**
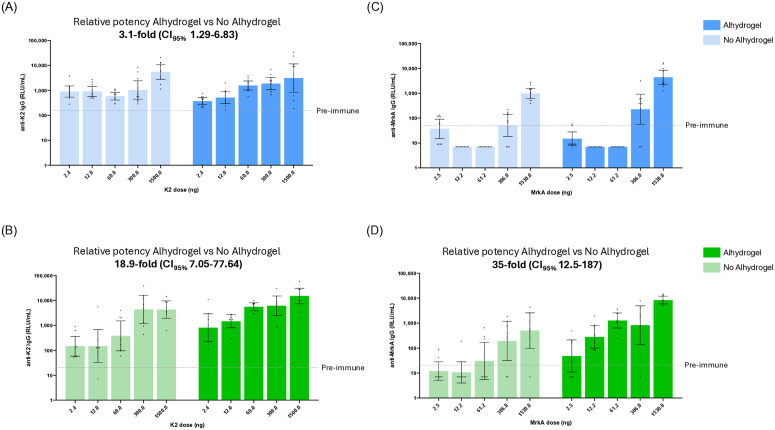
Dose-ranging studies with K2-Rhavi-FlaBD2-MrkA MAPS (530 kDa K2, 1.7 protein/saccharide molar ratio) in mice (A and C), and rabbits (B and D). Graphs showing Day 42 Luminex results for each formulation, and the relative potency of Alhydrogel vs No Alhydrogel formulation: **(A)** and **(B)** for anti-K2 IgG, **(C)** and **(D)** for anti-MrkA IgG. Bars in the graph represent geometric means of RLU/mL of each group and each dot results from each single animal. MrkA dose was calculated considering that it represents 34% in weight (MW MrkA (19,334 Da)/MW fusion construct (57,250 Da) of Rhavi-FlaBD2-MrkA.

In mice, two weeks after two immunizations, the anti-K2 specific IgG response was higher for formulations with Alhydrogel: in the absence of Alhydrogel, a three times higher dose of K2 MAPS complex was needed to reach the same response compared to the adjuvanted formulation (3.1 folds, CI_95%_ 1.3-6.8). Responses after immunization with 2.4 and 300 ng in absence of adjuvant were not significantly different from the pre-immune response. The linear dose-response range for the formulation without Alhydrogel was from 60 to 1500 ng, while for the formulation with Alhydrogel was from 12 to 300 ng (Fig 2A).

Also in rabbits, presence of Alhydrogel enhanced the anti-K2 IgG response, with a relative potency of the Alhydrogel *vs* no-Alhydrogel formulations of 18.9 folds (CI_95%_ 7.1-77.6). Only the first two lowest doses (with 2.4 and 12 ng) without Alhydrogel were not significantly different from the pre-immune IgG response. The linear dose range for Alhydrogel formulation was 2.4-60 ng, while it was 12–300 ng for no-Alhydrogel formulation (Fig 2B).

K2-MAPS was also tested in rats. Formulation without Alhydrogel was tested at 300 ng of K2, while the same doses used in mice and rabbits were tested with Alhydrogel. Also in this case, Alhydrogel increased the IgG responses (GeoMean fold increase of 5.8, S4 Fig). Anti-K2 IgG response after immunization with less than 300 ng dose, in presence of Alhydrogel, was not significantly different from pre-immune sera. At 300 ng dose a high number of non-responders was observed: 6 at day 42 in presence of Alhydrogel, all non-responders in absence of Alhydrogel (S4 Fig). A dose-response range was detected above 60 ng K2 dose, and likely the response did not reach a plateau at the 1500 ng highest dose tested (S4 Fig).

In mice, anti-MrkA IgG levels were low: without adjuvant, only the highest dose (1530 ng) produced a significant response, while with Alhydrogel, a significant response appeared at 306 ng (Fig 2C). The slopes of the two dose-response curves (Alhydrogel vs. No Alhydrogel) were significantly different. Anti-MrkA IgG response was significantly higher in the presence of Alhydrogel, when comparing formulations at the two highest doses tested (*p* of 0.0015 at 1530 ng MrkA and of 0.0110 at 306 ng of MrkA).

In rabbits, all groups, except for the two lowest dose groups in absence of Alhydrogel, elicited an anti-MrkA IgG response significantly different from the pre-immune sera. The linear dose response range was 2.5-61.2 MrkA ng with Alhydrogel and 12.2-306 ng without Alhydrogel. Also in this animal species, the presence of Alhydrogel enhanced anti-MrkA IgG immune response (35 folds, CI95% 12.5-187, Fig 2D).

In rats, the linear dose-response range for anti-MrkA IgG was 61.2-1530 MrkA ng in presence of Alhydrogel (S3 Fig). No statistically significant difference was evidenced in the anti-MrkA IgG response elicited by MAPS complex in presence or absence of Alhydrogel, when tested at 306 ng of MrkA (*p* of 0.0544); the ratio between the two geometric means was 11.4 (S3 Fig).

**Fig 3:**
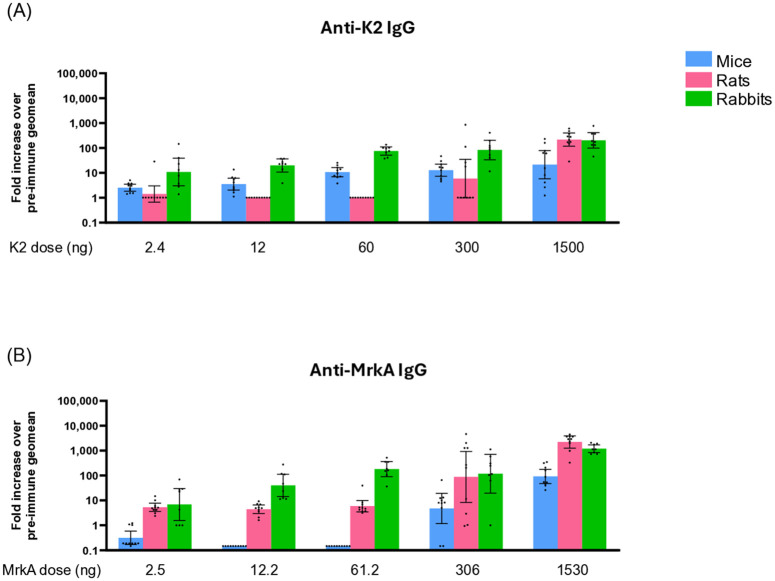
Comparison of immune response elicited by Alhydrogel-adjuvanted K2-Rhavi-FlaBD2-MrkA- MAPS complex at five increasing doses tested in mice, rats and rabbits. Results are expressed as fold increase with respect to the pre-immune geomean (dots relative to single animals and bars represent the geomean of the different groups). **(A)** Anti-K2 IgG response; **(B)** Anti-MrkA IgG response.

Fig 3 compares the anti-K2 and anti-MrkA IgG responses from the dose-ranging studies in the three animal species immunized with K2-Rhavi-FlaBD2-MrkA in presence of Alhydrogel, expressed as fold increase over the pre-immune levels. At the highest dose, rabbits and rats showed increases of similar magnitude in both anti-K2 and anti-MrkA IgG responses, whereas mice consistently exhibited lower fold increases than rabbits.

Sera from the two highest dose groups (300 and 1500 K2 ng), either with or without Alhydrogel were assayed by SBA assay against Kp K2 strain IL02364192, a clinical isolate from South Africa. Mice and rats sera showed no bactericidal killing, while rabbits sera elicited statistically significant higher IC50 values compared with pre-immune sera (S5 Fig). No differences were observed between Alhydrogel *vs* no Alhydrogel formulations at the doses tested (S5 Fig).

### Generation of K2 MAPS complexes with different structural characteristics

**Fig 4:**
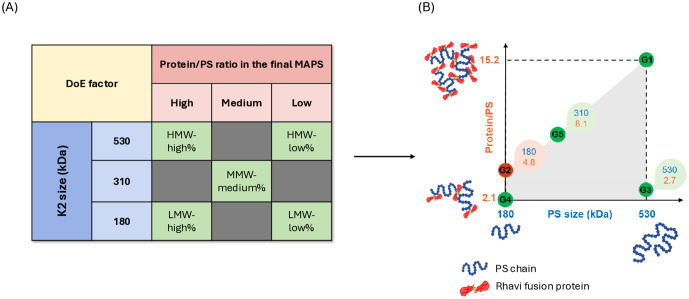
K2-Rhavi-FlaBD2-MrkA MAPS constructs produced in the study, deriving from the combination of two factors: the K2 size and the protein/polysaccharide ratio. **(A)** Table reporting the 5 different MAPS generated and tested in 5 different groups of rabbits. **(B)** Representation of the space defined by the actual characteristics of the produced MAPS (grey area).

To understand the impact on the immunological response elicited by MAPS complexes differing for K2 molecular weight (MW) and protein/polysaccharide ratio, MAPS constructs were generated from different combinations of these two factors (Fig 4A). Two different sizes of K2, hereinafter referred to as the HMW (High MW) and the MMW (Medium MW), respectively, were obtained by extracting the polysaccharide from bacteria by boiling in water or acetic acid respectively. The MW of the resulting K2 was 530 kDa, and 310 kDa, respectively. The O-acetylation level of the two polysaccharides was comparable (~55% vs ~ 45%, respectively) [34]. The MMW K2 was then used to produce a Low MW (LMW) polysaccharide, lowering the size to 180 kDa. Preliminary tests using the HMW K2 were performed to identify conditions to reach three different levels of polysaccharide biotinylation (high, medium, low), by changing CDAP concentration during the step of derivatization with biotin (S1 Table).

MAPS constructs were produced combining Rhavi-FlaBD2-MrkA with the differently biotinylated K2 of different sizes (Fig 4 and Table 1). These constructs, as expected, had different protein/polysaccharide w/w ratio, as direct consequence of the different polysaccharide biotinylation, ranging from 2.1 of the MAPS with LMW K2 (180 KDa) and low biotinylation level (1.5%), to 15.2 of the MAPS with HMW K2 (530 KDa) and high biotinylation level (7.9%). Protein/polysaccharide RU molar ratio of the final purified MAPS was, as expected, in good agreement with the % of biotinylated polysaccharide RU (Table 1). The MAPS, synthesized with the LMW K2 containing high biotinylation levels (LMW-high%, Table 1; highlighted by the red circle in Fig 4B), had a lower protein/polysaccharide w/w ratio compared to the corresponding MAPS with the HMW K2 (HMW-high%, Table 1) synthesized in the same conditions. Indeed, with the same CDAP concentration, the percentage of biotinylation per RU was lower for the LMW polysaccharide (Table 1). LMW K2 in general had lower biotinylation levels compared to HMW K2 (1.5% vs 3.1% and 3.4% vs 7.9%). In all these MAPS complexes, except for the HMW-low%, the rhavi subunit:biotin (mol:mol) was ~ 2, meaning that only one of the two rhavi subunits is involved in the binding of biotin (Table 1). The size (Z-ave) of the MAPS complexes was mainly dependent on the size of the polysaccharide, thus HMW K2 formed MAPS with bigger size compared to MMW and LMW K2 MAPS. With same K2 MW, MAPS complex with higher protein loading had bigger size (Table 1).

**Table 1:** Characterization of K2-Rhavi-FlaBD2-MrkA MAPS complexes differing for polysaccharide chain length and protein loading.

K2-Rhavi-FlaBD2-MrkA MAPS complex (polysaccharide MW-% biotinylation)	% biotinylated K2 RU	Mole protein/mole K2 RU ratio *	Rhavi subunit:biotin mol:mol	Protein/K2 w/w ratio	MrkA/K2 w/w ratio**	Z-Ave (d, nm)
**HMW-high%**	7.9	0. 088	2.2	15.2	5.2	78.5
**LMW-high%**	3.4	0.028	1.6	4.8	1.6	48.1
**HMW-low%**	3.1	0.016	1.0	2.7	0.9	65.2
**LMW-low%**	1.5	0.012	1.6	2.1	0.7	34.9
**MMW-medium%**	5.1	0.047	1.8	8.1	2.8	46.0

An example of calculations reported in this Table is in S1 Text.

* Rhavi-FlaBD2-MrkA (as dimer) moles per mole of polysaccharide RU.

** MrkA amount in weight in Rhavi-FlaBD2-MrkA corresponds to the 34% (MW MrkA (19,334 Da)/MW fusion construct (57,250 Da) of the total protein.

### Comparing MAPS complexes differing for K2 length and protein to saccharide ratio in rabbits

**Fig 5:**
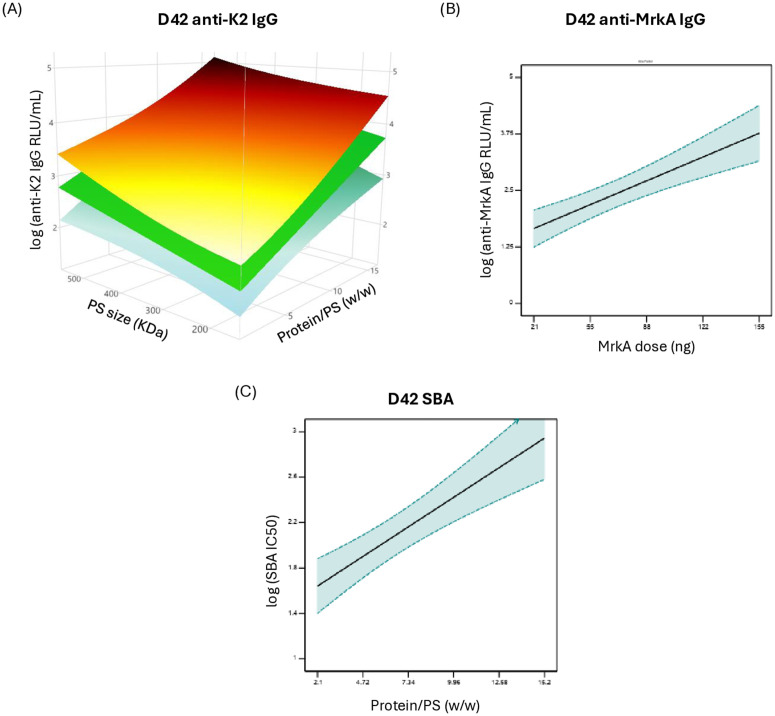
Effect of polysaccharide length and protein to saccharide ratio on K2-Rhavi-FlaBD2-MrkA MAPS complex immunogenicity. **(A)** Graph showing the model with 95% confidence intervals obtained for the anti-K2 IgG response at day D42. **(B)** Graph showing the model for the anti-MrkA IgG response at day 42. **(C)** Graph showing the model for the SBA IC50 at day 42. 95% confidence intervals are indicated by dotted lines.

Based on previous results, rabbits were selected as preferred animal model to conduct the study to evaluate impact of MAPS features on immunogenicity. Animals were immunized intramuscularly at day 0 and day 28 with five K2-Rhavi-FlaBD2-MrkA MAPS complexes differing in K2 size and protein loading (Fig 4A) at a K2 dose of 30 ng, thus within the linear range found in the previous dose-ranging study, enabling the identification of possible differences in the immunogenicity elicited by the tested MAPS. All constructs, except for LMW-low% MAPS complex, elicited a significant anti-K2 IgG response after first vaccination (S2 Table). The response further increased post second injection, except for the LMW-high% MAPS (S6A Fig and S3 Table). The anti-MrkA IgG responses induced by Rhavi-FlaBD2-MrkA MAPS complexes with low and medium protein loading were not different from the pre-immune sera after first injection (S4 Table). A boost in the anti-MrkA IgG response was observed after second injection, except for the LMW-low% MAPS (S6B Fig and S3 Table). Applying regression analysis to IgG responses at day 42, we found that both polysaccharide length and protein/polysaccharide ratio have an impact on anti-K2 IgG response, which increases with higher polysaccharide length and higher protein/polysaccharide ratio, with no significant interaction between the two parameters (Fig 5A). The model showed non-significant lack-of-fit (*p* of 0.0835). Anti-MrkA IgG response was not dependent to any of these factors or their interaction, and it increased only with the dose of MrkA (Fig 5B). Also in this case the model had non-significant lack-of-fit (*p* of 0.2245). Same outcomes have been obtained by modeling anti-K2 and MrkA IgG responses at day 27 (S7 Fig). SBA measured on day 42 sera was dependent only from protein/polysaccharide ratio (Fig 5C, non-significant lack-of-fit model with a *p* of 0.6194, S7 Fig).

When we included MAPS complex size (Z-ave) as predictor, this factor was redundant, being in fact an attribute derived from polysaccharide size and protein/polysaccharide ratio.

### Comparing MAPS complexes differing for carrier protein

**Fig 6:**
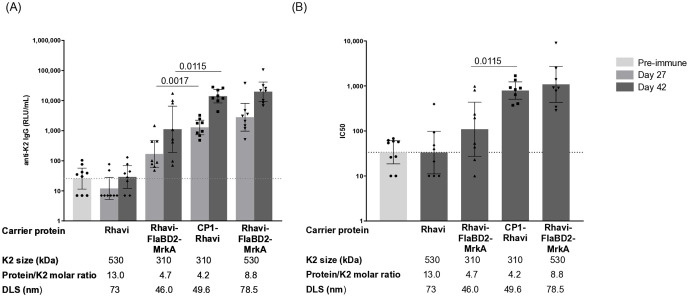
Immunogenicity induced in rabbits by MAPS complexes using different proteins as carrier for K2. **(A)** Anti-K2 IgG response. **(B)** SBA against Kp K2 strain IL02364192 expressed as IC50. Bars in the graph represent geometric means of RLU/mL of each group and each dot results from each single animal.

Another factor that could impact MAPS complex immunogenicity is represented by the nature of the protein. CP1-rhavi and Rhavi were compared to Rhavi-FlaBD2-MrkA as carrier proteins. For its small size, ~ 31 kDa, Rhavi would allow an easier MAPS complex purification from excess of protein. CP1-Rhavi is a fusion of two pneumococcal proteins derived from genetically conserved surface proteins, has been already included in MAPS assemblies tested as candidate vaccine against *Streptococcus pneumoniae* [23]. The CP1-rhavi MAPS complex was synthesized adopting the specific conditions of the central point of the previous design scheme (Fig 4A), thus using a MMW polysaccharide with a medium biotinylation level. The protein/polysaccharide RU molar ratio of CP1-rhavi MAPS complex (0.042) resulted to be close to that of the Rhavi-FlaBD2-MrkA MAPS complex (0.047) produced with the same biotinylated polysaccharide. Rhavi, having a smaller size compared to the other two fused proteins, was instead assembled to a HMW K2 with a high % of biotinylation. This resulted in a high protein/polysaccharide molar ratio (0.13) (Fig 6). Also in this case, the size of the MAPS complex was mainly driven by the size of the polysaccharide.

When tested *in vivo*, CP1-rhavi MAPS complex induced a significantly higher level of anti-K2 IgG antibodies compared to the corresponding Rhavi-FlaBD2-MrkA MAPS complex, at both day 27 and day 42 (*p* of 0.0017 and 0.0115, respectively, Fig 6A and S5 Table). There was no difference (*p* of 0.1521 and 0.3810 at day 27 and day 42, respectively) between the CP1-rhavi MAPS complex and the best performing Rhavi-FlaBD2-MrkA MAPS complex from the previous study (HMW-high%, Fig 6A and S5 Table). Rhavi alone did not perform well as carrier for K2: the corresponding MAPS construct did not elicit a significant anti-K2 IgG response (Fig 6A). SBA analysis on day 42 sera was consistent with the ELISA results, with only CP1-rhavi and the HMW-high% K2-Rhavi-FlaBD2-MrkA MAPS complexes sera promoting bactericidal activity against K2 isolate IL02364192 (Fig 6B). CP1-rhavi performed better as carrier protein compared to Rhavi-FlaBD2-MrkA also in mice and rats (S8 Fig).

### Protection in a mouse challenge model

**Fig 7:**
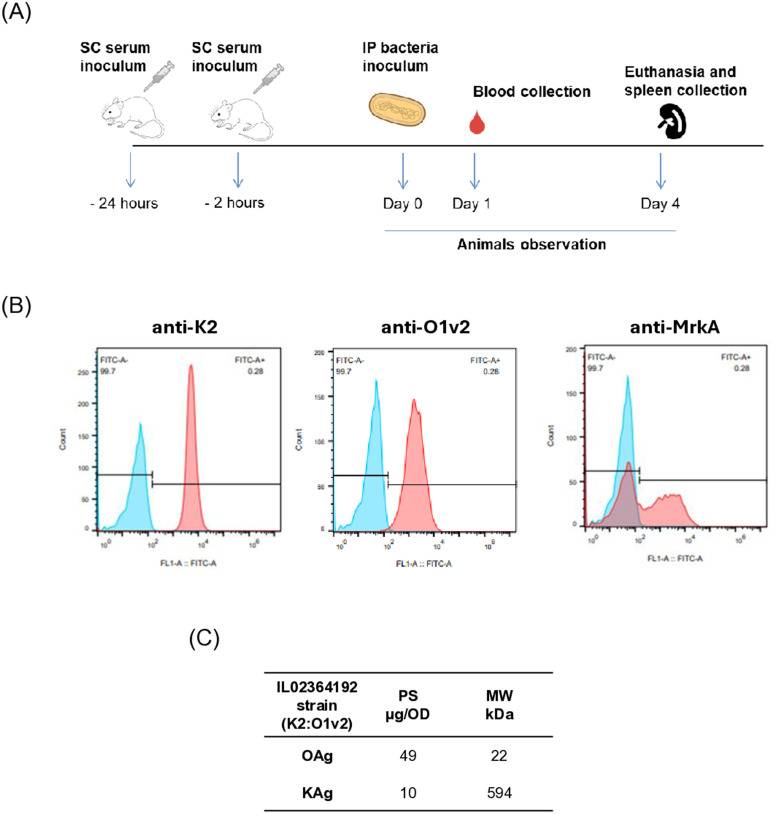
Mice challenge with Kp clinical isolate IL02364192. **(A)** Challenge scheme. **(B)** Characterization of Kp strain in the condition of the challenge by flow cytometry. **(C)** Relative abundance and size of the OAg and KAg of Kp inoculum expressed as µg of polysaccharide per bacteria optical density (OD).

To show protective ability of antisera generated by the most promising constructs, rabbit sera raised against K2-CP1-rhavi MAPS complex and the most immunogenic K2-Rhavi-FlaBD2-MrkA MAPS complex were pooled and passively transferred to mice before intraperitoneal challenge with the same Kp clinical isolate used in SBA (IL02364192, K2:O1v2), according to the scheme reported in Fig 7A. KAg and O-Antigen (OAg) expression in the bacterial inoculum was verified by flow cytometry, confirming the presence of a population positive for MrkA expression (Fig 7B). The amount of the two sugars was also quantified, and a higher abundance of the OAg (22 kDa) over the KAg (594 kDa) was found (OAg/KAg ratio of ~ 5) (Fig 7C).

**Fig 8:**
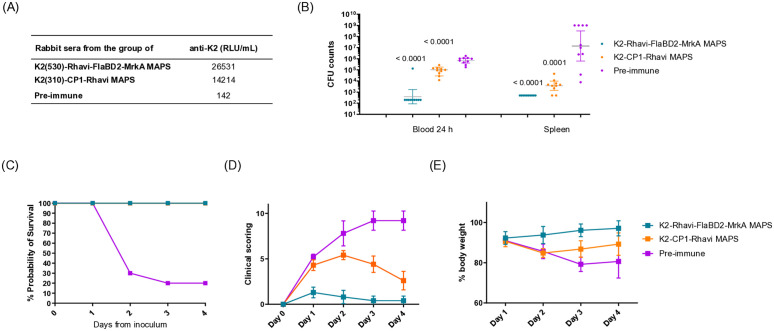
*In vivo* protection of MAPS complexes rabbit sera. **(A)** Table reporting the pooled rabbit sera used for the passive transfer in mice and related anti-K2 IgG. **(B)** CFU counts in blood 24 h after challenge and in the collected spleens at the completion of the study with Lognormal Welch T test *vs* pre-immune. **(C)** Probability of survival plot. **(D)** Mean clinical observation score. **(E)** Mean group % body weight.

Antibodies from the two MAPS constructs conferred complete protection, compared to 20% protection observed with pre-immune sera four days post-challenge (Fig 8C). Colony forming units (CFU) counts in the blood (24 hours post-infection) and spleen (at the end of the study) were assessed, revealing significantly lower bacterial loads in both blood and spleens of mice treated with anti-K2-Rhavi-FlaBD2-MrkA or anti-K2-CP1-rhavi MAPS complexes antibodies *vs* those receiving pre-immune sera (*p* < 0.0001 for both MAPS in blood and spleen) (Fig 8B and S6 Table). Moreover, anti-K2-Rhavi-FlaBD2-MrkA MAPS complex sera seemed to more efficiently promote bacterial clearance from the blood and spleen compared to CP1-rhavi (*p* of 0.0003 and 0.0007, respectively).

The weight loss (Fig 8E) and behavioural/clinical observations of each animal were combined into a clinical score for each animal. The group mean scores were plotted versus time post challenge (Fig 8D). A clinical score of 10 was assigned to mice that were sacrificed. The animals that succumbed to the challenge exhibited multiple signs of infection. These included ruffled fur, arched posture, eye inflammation and immobility. Lower scores were measured for all mice treated with anti-K2-Rhavi-FlaBD2-MrkA MAPS complex sera compared to mice receiving anti-K2-CP1-rhavi MAPS complex sera or pre-immune sera.

## Discussion

In this study we evaluated the MAPS platform, combining polysaccharide antigens to conserved, pathogen specific proteins, as an approach to develop polysaccharide-based vaccines against Kp, a pathogen characterized by high capsular glycan variability. Kp is a leading cause of neonatal sepsis in LMICs and is included on the WHO list of priority AMR pathogens [7], highlighting the urgent need for new preventive strategies. To tackle neonatal sepsis, a maternal immunization strategy could be particularly valuable: passive transfer of maternal antibodies would provide newborns with a repertoire of antigen-specific IgG during the critical postnatal period, with an antibody half-life of approximately 28–35 days [35]. Recent clinical experience with a hexavalent glycoconjugate vaccine against Group B *Streptococcus* showed that vaccination during pregnancy can elicit antibodies that reach protective levels in infants [36].

We selected the K2 capsular polysaccharide as a model antigen because it is among the serotypes most commonly associated with neonatal sepsis in LMICs [30]. Kp MAPS complexes were generated by incorporating MrkA [28], a highly conserved Kp putative antigen [37,38]. Our goals were to generate and thoroughly characterize a panel of MAPS constructs, to identify manufacturing and design parameters that influence immunogenicity, and to define guidance for constructing an optimal MAPS based Kp vaccine. Given the unknown translability of animal results in humans, we assessed immunogenicity in parallel in mice, rats and rabbits. On the manufacturing side, we compared two activation chemistries for polysaccharide biotinylation, CDAP and EDAC, and conducted accelerated stability studies on the biotinylated intermediates. Both chemistries produced intermediates that were stable at 4 °C and 25 °C for 21 days; only minor declines in biotinylation were observed at 40 °C and were similar for both methods. We selected CDAP chemistry not because it provided superior stability, but because it offers practical advantages: a faster, simpler single-step reaction that operates efficiently at pH 9, according to an optimized procedure described by Nappini et al. [33], and a linear relationship between CDAP concentration and biotinylation degree. This linearity enables controlled production of polysaccharides with defined biotinylation levels and facilitates extension of the workflow to other Kp capsular polysaccharides, where chemistry selection can be tailored to polysaccharide structure and composition. The approach followed in this work can be rapidly extended to other KAg, to verify stability of the biotinylated intermediate and select the most appropriate chemistry for the specific polysaccharide structure and composition.

Immunologically, dose-ranging studies demonstrated that K2-Rhavi-FlaBD2-MrkA MAPS construct induce strong anti-K2 IgG even at very low polysaccharide doses (<1 µg). Rabbits generated especially robust responses, with a linear dose range between ~2.4 and 60 ng of polysaccharide when Alhydrogel was included. Alhydrogel significantly potentiated MAPS complex immunogenicity across species and also increased anti-MrkA response. Measurable SBA was detected principally in rabbit sera, which further motivated selecting rabbits for more detailed optimization experiments. Rabbits are commonly used for testing vaccine reactogenicity [39], and also as a reliable animal model for many infectious diseases [40].

Using a DoE-style experimental design and regression analysis, we systematically varied polysaccharide molecular weight and protein ratio to identify drivers of anti-polysaccharide and anti-protein responses. By immunizing with a dose on the linear portion of the dose-response curve, we were able to create an antibody response surface with K2 MW and protein ratio as the inputs. This approach represents a rational roadmap for developing polysaccharide-based vaccines (e.g., MAPS and glycoconjugates), allowing to identify optimal design by understanding impact of each potential critical parameter and their interactions on the corresponding immune response [33]. Two main findings emerged. First, higher protein loading and higher molecular weight (HMW) polysaccharide both positively influenced the anti-K2 immune response across the tested design space. Second, anti-MrkA IgG was primarily dependent on the absolute MrkA dose and was not substantially affected by polysaccharide size or MAPS composition. From a manufacturing perspective, the favorable effect of HMW native polysaccharide is advantageous because it eliminates the need for saccharide fragmentation prior to biotinylation, as usually required for traditional glycoconjugates; however, increasing percent protein loading has cost implications because it requires larger protein quantities for assembly.

We also observed that carrier protein identity matters. For traditional glycoconjugates, not all proteins work as effective carriers: different proteins can actively shape both the magnitude and the quality of the anti-polysaccharide antibody response [41]. CP1-rhavi induced in rabbits significantly higher level of anti-K2 antibodies when compared to Rhavi-FlaBD2-MrkA in complex with the same biotinylated polysaccharide. CP1-rhavi was also more bactericidal, with the same trend observed in mice and rats. Conversely, sera from the most immunogenic Rhavi-FlaBD2-MrkA MAPS complex provided better protection than sera from CP1-rhavi MAPS, after passive transfer to mice and challenge with a Kp clinical isolate. Although both sera prevented mortality, mice that received anti‑Rhavi-FlaBD2-MrkA sera exhibited better clinical scores and significantly lower bacteremia at 24 hours post‑infection, as well as reduced splenic bacterial counts at the end of the study. This could imply a protective role of anti-MrkA antibodies against Kp, in accordance with published data [26,42,43]. Protective role of MrkA could happen through different mechanisms from SBA, considering that CP1-Rhavi and Rhavi-FlaBD2-MrkA MAPS complexes elicited similar SBA titers. It has been reported that an active immunization with recombinant MrkA did not protect mice in a sepsis model with a lethal dose of hyper capsulated K1 strain [44]. A combination of anti-MrkA and anti-polysaccharide antibodies might be necessary to confer protection against highly virulent isolates with increased capsule production.

Overall, this work makes several concrete contributions to MAPS vaccine development for Kp. We demonstrated a robust and scalable CDAP biotinylation workflow, identified key design parameters (polysaccharide molecular weight and protein loading) that predictably modulate anti-polysaccharide responses, and highlighted that protein carrier choice can shape both antibody quantity and *in vivo* protective quality. These findings are broadly applicable to other Kp capsular serotypes and provide rational design principles for constructing optimized, multicomponent MAPS complexes formulations.

This study has limitations. Translating animal findings to humans remains uncertain and will require careful clinical evaluation, particularly in the context of maternal immunization where safety, transplacental antibody transfer kinetics, and infant protection need to be characterized. SBA is a meaningful functional assay but does not capture all potential protective mechanisms; complementary assays (e.g., opsonophagocytic killing, neutralization of adhesins or biofilm-related functions) would help clarify how anti-MrkA antibodies mediate protection. Finally, the production cost-benefit balance of increasing protein loading versus leveraging native HMW polysaccharide deserves further evaluation in scale-up and manufacturing studies.

In conclusion, our results support further development of MAPS as a platform for *K. pneumoniae* vaccines. The work identifies actionable manufacturing and design levers-activation chemistry, polysaccharide size, protein loading, and carrier selection-that can be applied across capsular types and inform the rational design of MAPS multivalent vaccines, including formulations intended for maternal immunization to protect neonates from invasive Kp disease.

## Materials and methods

### Ethics statement

GSK is committed to the Replacement, Reduction and Refinement of animal studies (3Rs). Non-animal models and alternative technologies are part of our strategy and employed where possible. When animals are required, the application of robust study design principles and peer review minimizes animal use, reduces harm and improves benefit in studies.

Mice and rabbits immunogenicity studies were performed at Charles River Laboratories (France) and were authorized by the French Ministry of Higher Education and Research (Ministère de l’Enseignement supérieur et de la Recherche; MESR) under APAFIS project codes APAFIS #43004–2023041910185277 v4 (mice; authorization date: 04 May 2023) and APAFIS #39244–2022110910396896 v3 (rabbits; authorization date: 19 April 2023). The projects received a favorable opinion from the Animal Experimentation Ethics Committee no. 041 (comité d’éthique en expérimentation animale n°041) and were carried out in compliance with animal welfare standards according to European Directive 63/2010, the local legislation, and the GSK policy on the Care, Welfare and Treatment of Animals. Rats immunogenicity study was performed at GSK animal facility under the animal project 817/2024-PR, approved by the Italian Ministry of Health. Mice challenge study was performed at Fondazione Toscana Life Science Animal Care Facility under the animal project 8/2025-PR, approved by the Italian Ministry of Health.

### K2 isolation, purification and characterization

Kp strains NCTC11228 (K2:O1v1) acquired from Public Health England were plated on Worfel-Ferguson Agar plates (yeast extract 2 g/L, MgSO_4_·7H_2_O 0.25 g/L, K_2_SO_4_ 1 g/L, NaCl 2 g/L, and sucrose 20 g/L) [45] and incubated ON at 30 °C. After growth, the bacterial biomass was collected with a solution of AcOH 1% (*v*/*v*) in water, and the suspension was incubated for 6 h at 100 °C in a preheated thermoblock heater. The material was then centrifuged (3,369 rcf, 30 min, 4 °C), and the polysaccharide-containing supernatant was collected and filtered with 0.22 µm membranes. The bacterial supernatant, resolubilized in water after lyophilization, was purified through fractional precipitation with CTAB as previously reported [34]. K2 was characterized by High-performance liquid chromatography–Size Exclusion Chromatography (HPLC–SEC) with dRI detection to estimate the molecular size distribution, using TSK gel G6000 and G3000 PWXL columns connected in series (respectively 30 cm x 7.8 mm; particle size 13 µm; cod. 808023 and 30 cm x 7.8 mm; particle size 7 µm; cod. 808021) with TSK gel PWXL guard column (4.0 cm x 6.0 mm; particle size 12 µm; cod.808033) (Tosoh Bioscience) using as mobile phase 0.1 M NaCl, 0.1 M NaH_2_PO_4_, 5% ACN, pH 7.2 at a flow rate of 1 mL/min. K2 peak molecular mass (MP: mode of population) was calculated using pullulans as standards in the range 23 – 800 kDa. K2 quantification was based on glucose quantification through High-performance Anion-exchange Chromatography with Pulsed Amperometric Detection (HPAEC–PAD) analysis with commercial sugar monomer calibration curve (0.5–10 µg/mL) following acid hydrolysis at 100 °C for 4 h in 2 M trifluoroacetic acid (TFA) [46], using for the separation a CarboPac PA1 column (2 × 250 mm) coupled with a PA1 guard column (2 × 50 mm) (Thermo Fisher Scientific, Waltham, MA, USA). DNA and protein impurities were determined by measuring absorbance at 260 nm and through microBCA, using bovine serum albumin (BSA) as a reference following the manufacturer’s instructions (Thermo Fisher Scientific), respectively. ^1^H NMR spectroscopy was used to confirm K2 identity and purity and to measure O-acetylation level.

### Production of K2 with different lengths

Three different K2 sizes (HMW, MMW and LMW) were targeted to generate a panel of K2- Rhavi-FlaBD2-MrkA MAPS differing for saccharide length. HMW and MMW polysaccharide were obtained using different extraction conditions of the polysaccharide from bacteria prior purification: the HMW K2 was extracted incubating bacteria resuspended in water at 100 °C for 6 h. The MMW K2 originated from an acetic acid extraction (1% AcOH v/v, 100 °C, 6 h). The LMW K2 was instead produced starting from the MMW K2, performing 30 cycles of sonication (VibraCell, Sonics and Materials Inc.), after placing a diluted polysaccharide solution in an ice bath. The sonication cycle consisted of 30 sec of pulses followed by 30 sec of rest. After sonication, K2 molecular size distribution was measured using TSK gel G6000 (30 cm x 7.8 mm; particle size 13 µm; cod. 808023) and G3000 PW_XL_ (30 cm x 7.8 mm; particle size 7 µm; cod. 808021) columns in series with a TSK gel PW_XL_ guard column (4.0 cm × 6.0 mm; particle size 12 µm; cod. 808033) (Tosoh Bioscience). Buffer 0.1 M NaCl, 0.1 M NaH_2_PO_4_, 5% ACN, pH 7.2 was used as mobile phase at a flow rate of 1 mL/min. Pullulans (in the 2,000 –50 kDa range) were used to build a standard calibration curve. Structural integrity and O-acetylation level were checked via ^1^H NMR spectroscopy.

### Synthesis, purification and characterization of MAPS complexes

#### K2 biotinylation step.

K2 polysaccharide was activated with CDAP and subsequently derivatized with Amine-PEG3-Biotin linker (Thermo Fisher Scientific). Briefly, K2 was solubilized at 1 mg/mL in 100 mM DABCO pH 9.2 and was placed in an ice bath. Reaction with CDAP (0.82, 0.37 or 0.16 mg/mL, to provide a high, medium or low polysaccharide activation, respectively) was performed at 0 °C for 15 min under stirring, as previously reported [33]. After 15 min, Amine-PEG3-Biotin linker solubilized in 0.1 M DABCO pH 9.2 (40 mg/mL) was chilled and added to a final concentration of 2.5 mg/mL. Reaction mixture was kept in agitation for 5 h at room temperature (RT). The quenching of the reaction was performed adding 1:1 v/v 1 M Glycine in 100 mM sodium phosphate pH 7.2; the mixture was then kept ON a 4 °C. Biotinylated polysaccharides were purified through a first G25 column (HiPrep 26/10 Desalting, Cytiva) against 1 M NaCl followed by a second run against water, to remove excess of free linker.

EDAC/S-NHS chemistry was also tested for polysaccharide derivatization with biotin. In the following order, S-NHS (15 g/mL) and EDAC (11 mg/mL) were added to K2 (2 mg/mL) in 100 mM 2-(N-morpholino) ethanesulfonic acid (MES), 150 mM NaCl, pH 5.4, and the reaction was incubated for 30 min at RT. After this time, Amine-PEG3-Biotin linker solubilized in 100 mM MES 150 NaCl pH 5.4 (40 mg/mL) was added to a final concentration of 2.7 mg/mL. The pH was adjusted to 7.4 with 1 M Sodium Bicarbonate pH 7.3 in the first 4 hours, leaving then the reaction mixture in agitation ON at RT.

Polysaccharide biotinylation level (express as molar ratio between biotin and polysaccharide RU) was quantified using Pierce Fluorescence Biotin Quantitation Kit (Thermo Fisher Scientific) or by HPLC-SEC (214 nm UV absorbance) through the indirect quantification of the excess of unbound Streptavidin (SA) after the assembly reaction between a defined amount of SA and the biotinylated polysaccharide performed directly in the HPLC sample vials. The SA calibration curve was in the range 0.5-5.8 nmol/mL. The analyte quantification was performed using TSK gel 3000PW_XL_ column (30 cm x 7.8 mm; particle size 7 µm; cod. 808021) with a TSK gel PW_XL_ guard column (4.0 cm × 6.0 mm; particle size 12 µm; cod. 808033) (Tosoh Bioscience) and 0.1 M NaCl, 0.1 M NaH_2_PO_4,_ 5% ACN, pH 7.2 buffer was used as mobile phase at a flow rate of 0.5 mL/min.

#### Synthesis of MAPS complexes.

Rhavi, Rhavi-FlaBD2-MrkA [28] and CP1-rhavi [47] fusion proteins were provided by GSK Technical Research and Development.

MAPS complexes were assembled incubating the biotinylated polysaccharide with an excess of protein with a biotin binding site equal to 4-times the moles of biotinylated polysaccharide RU (biotinylated RU to rhavi dimeric protein 1:2 mol/mol) at 4 °C ON under gentle mixing on a rocker. MAPS complexes were purified through a SEC with HiPrep 16/60 Sephacryl S300 HR column (Cytiva) to remove excess of free protein. Isocratic elution at 0.5 mL/min in PBS was used.

Purified MAPS complexes were characterized by HPAEC–PAD analysis for sugar quantification [46] and by microBCA for protein quantification, using BSA as a reference following the manufacturer’s instructions (Thermo Fisher Scientific); the ratio of protein to saccharide was then calculated. In S1 Text we reported an example for the calculation of mole protein/mole K2 RU ratio. Dynamic Light Scattering (DLS) was used to determine the size of MAPS constructs. DLS measurements were performed with a Zetasizer Nano ZS (Malvern, Herrenberg, Germany). ZS Xplorer software used to collect and analyze data. Each MAPS sample (protein concentration range 40 – 600 μg/mL) was analyzed using single-use polystyrene microcuvette (ZEN0040, Alfatest) with a path length of 10 mm, setting temperature at 25 °C. The hydrodynamic diameter (in nanometers) was expressed by a Z-average value (general purpose algorithm) of three measurements for each sample, providing also a polydispersity index of the size values calculated.

### Immunogenicity studies in animal models

MAPS complexes were diluted with 2% Sucrose and 0.02% Tween 80, to limit eventual aggregation phenomena, in 10 mM phosphate pH 6.5, to better preserve the O-acetylation level. Dose ranging studies were performed in mice, rats and rabbits. 10 4–6 weeks old female BALB/c mice per group were subcutaneously immunized with 200 μL of K2-Rhavi-FlaBD2-MrkA MAPS complex on day 0 and 28 in presence (0.125 mg Al^3+^ per dose) or absence of Alhydrogel. Sera were collected on day 42.

Female New Zealand White rabbits (8 per group, of at least 12 weeks old) and female Sprague Dawley rats (10 per group, of at least 8 weeks old) were intramuscularly immunized with 500 μL and 200 μL of K2-Rhavi-FlaBD2-MrkA MAPS complex, respectively, in presence (0.125 mg Al^3+^ per dose) or absence of Alhydrogel, using the same immunization scheme of the mouse study.

Based on results from dose ranging studies, impact of saccharide length, polysaccharide to protein ratio, MAPS complex size and carrier protein was further evaluated in rabbits. Animals were immunized with different MAPS constructs at K2 dose of 30 ng, on days 0 and 28. Sera were collected on days -1, 27 and 42.

Individual animal sera were tested for anti-K2 and anti-MrkA total IgG by multiplex bead- based binding assay (Luminex) adapting a previously described method [48] (using biotinylated K2 and MrkA protein on beads). Results are reported as anti-antigen specific IgG Relative Luminex Unit (RLU)/mL obtained interpolating MFI against standard curve generated by diluting anti-antigen specific hyperimmune sera (8 points 3-fold serial dilutions), which was assigned an arbitrary value of 100 relative RLU/mL. Serum Bactericidal Activity (SBA) of sera against Kp K2 strain IL02364192 (K2:O1v2) was performed on day 42 sera, as previously reported [49,50]. Results were expressed as IC50, the reciprocal serum dilution resulting in a 50% growth inhibition of the bacteria present in the assay. GraphPad Prism software (GraphPad Software, La Jolla, CA, USA) was used for curve fitting and IC50 determination. A titer equal to half of the first dilution of sera tested [10] was assigned to sera not bactericidal.

### Passive transfer of polyclonal rabbit sera and challenge of mice

Groups of ten female CD1 mice, aged 9 weeks, subcutaneously received pooled polyclonal sera (0.4 mL 24 hours before and 0.2 mL 2 hours before challenge) derived from rabbits immunized with selected MAPS. From each group equal volumes from each rabbit were pooled before passive transfer. Pre-immune rabbit serum served as negative control. Two hours after the final serum dose, mice were challenged intraperitoneally with 1 × 10^6^ CFU of the clinical Kp isolate IL02364192. To prepare the inoculum, the strain was streaked onto LB agar and incubated at 37 °C ON; the following day five colonies were inoculated into 5 mL LB and grown ON at 37 °C with shaking. The ON culture was then diluted into 100 mL LB to an initial OD600 of 0.05 and incubated at 37 °C with shaking for ~1.5 hours until reaching ~0.5 OD600. Cells were pelleted by centrifugation for 10 minutes at 3,369 rcf at RT and resuspended in 50 mL PBS supplemented with 1% gastric mucin. Clinical signs were monitored daily after inoculation, and any adverse findings were recorded. Animals that met predefined humane endpoints were euthanized according to ethical requirements. CFU were counted in the blood 24 hours after the infection and in the spleen at the end of the study or immediately after euthanasia for animals that reached humane endpoints. An arbitrary titer of 10^7^ CFU/mL and 10^9^ CFU/mL were assigned to the blood or spleens of animals that were found dead. Protective efficacy of the different vaccine regimens was evaluated up to day 4 post-challenge.

### Data analysis

Statistical analysis was performed using GraphPad Prism 11 (GraphPad Software, La Jolla, CA, USA) under the assumption of log−normality [51,52], which was adopted to account for the biological variability typically observed in immunological measurements. For two−group comparisons, Welch’s t−tests assuming log−normal data distribution were performed; all P values and GMRs with 95% CIs are reported in Supporting information. Likewise, a paired t−test based on a log −normal data distribution was used to evaluate the responses induced by the same formulation at different timepoints.

CombiStats v.7 EDQM software was used for parallel line analysis (PLA). A logarithmic transformation of the response (ln(response)) and dose (ln(dose)) was performed. For each series, three consecutive doses (out of five) that yielded the steepest singular slope were selected. Subsequently, PLA was applied to calculate the common slope across the selected doses and the relative potency.

In the study that evaluated impact of MAPS constructs characteristics on the immune response, multiple regression analyses (ANOVA, Design Expert software-version: 13.0.11.0) were performed on the log-transformed RLU/mL (day 27 or day 42) and log-transformed SBA IC50 (day 42) in order to find the surface to correlate the log-transformed immunological data (IgG or SBA) with MAPS features (K2 length, MAPS size and K2 to protein ratio). The interpolation was performed considering a surface with the first order terms and all the interactions between them model. Subsequently, an iterative backward elimination (with a threshold of *p* < 0.05) of non-significant terms was applied. 3D surface graph with 95% confidence interval was realized with JMP 17.2.0 (JMP Statistical Discovery LLC, San Francisco, CA, USA).

## Supporting information

S1 Fig Kinetic modeling for CDAP (A) and EDAC (B) chemistry. Dots are the experimental nmol biotin/mg K2 (y-axis) values measured at selected time points (3, 6, 9, 13, 17 and 21 days, x-axis).
(TIF)

S2 Fig ANCOVA analysis on K2-biotin stability data. The effect of the different chemistry on the stability trend (Day_pool*Chemistry term) is not significant at all the three temperatures. Only at 40 °C a significant trend (*Day_pool* term) is found (p value of 0.009). *Chemistry* term is significant as the two starting nmol biotin/mg K2 in K2_CDAP_-biotin and K2_EDAC_-biotin are different.
(TIF)

S3 Fig K2-Rhavi-FlaBD2-MrkA MAPS SEC purification (A) and HPLC-SEC characterization (B). (A) SEC purification of MAPS complex with HiPrep Sephacryl S-300 HR column 16 mm × 600 mm (Cytiva). (B) HPLC-SEC (ACQUITY UPLC Protein BEH SEC Column, 200 Å, 1.7 µm, 4.6 mm × 150 mm, Waters) chromatogram of purified MAPS (black line) in overlay with Rhavi-FlaBD2-MrkA protein (blue line).
(TIF)

S4 Fig Dose-ranging study in rats with K2-Rhavi-FlaBD2-MrkA MAPS complex. Graphs showing anti-K2 specific (A) and anti-MrkA (B) specific IgG response.
(TIF)

S5 Fig SBA results on selected groups from mice (A), rabbits (B) and rats (C) dose-ranging studies with K2-Rhavi-FlaBD2-MrkA MAPS complex. In the left panel D42 anti-K2 IgG Luminex data are reported. No differences were observed between Alhydrogel vs no Alhydrogel formulations at the doses tested in rabbits (GMT of 482, 95% CI 284.6-815.7, and of 222, 95% CI 93.49-525.2, respectively, at 1500 ng; GMT of 411, 95% CI 194.6-871.4, and of 233, 95% CI 124.4-433.7, respectively, at 300 ng).
(TIF)

S6 Fig Immunogenicity induced in rabbits by MAPS constructs differing for K2 length and protein to saccharide ratio. (A) Anti-K2 specific and (B) anti-MrkA specific IgG response. Bars in the graph represent geometric means of RLU/mL of each group and each dot results from each single animal.
(TIF)

S7 Fig ANOVA for Day 27 and Day 42 anti-K2 and MrkA IgG response models and for D42 SBA model. (TIF)

S8 Fig Comparison between Rhavi-FlaBD2-MrkA and CP1-rhavi in mice (A) and rats (B). (A) D42 anti-K2 IgG response of K2-Rhavi-FlaBD2-MrkA MAPS (K2 530 kDa; protein/K2 w/w ratio of 3.0) and K2-CP1-Rhavi MAPS (K2 530 kDa; protein/K2 w/w ratio of 2.0) tested in mice at 300 ng of K2 with and without 0.625 mg/mL [Al^3+^] of Alhydrogel (SC immunization with 200 μL of vaccine at day 0 and day 28). (B) D42 anti-K2 IgG response of K2-Rhavi-FlaBD2-MrkA and K2-CP1-Rhavi MAPS tested in rats at 300 ng of K2 with 0.625 mg/mL [Al^3+^] of Alhydrogel (IM immunization with 200 μL of vaccine at day 0 and day 28). (C) Lognormal Welch T test between K2-Rhavi-FlaBD2-MrkA MAPS and K2-CP1-Rhavi MAPS.
(TIF)

S1 Table Targeting different polysaccharide biotinylation levels by changing CDAP concentration in reaction. (XLSX)

S2 Table Lognormal Welch T test between each group anti-K2 IgG of the DoE-style immunogenicity study in rabbits against pre-immune. (XLSX)

S3 Table Lognormal paired t-test between D27 and D42 IgG data of each group of the DoE-style immunogenicity study in rabbits. (XLSX)

S4 Table Lognormal Welch T test between each group anti-MrkA IgG of the DoE-style immunogenicity study in rabbits against pre-immune. (XLSX)

S5 Table Lognormal Welch T test between groups of the immunogenicity study in rabbits with MAPS complexes differing for the carrier protein. (XLSX)

S6 Table Lognormal Welch T test between each group of the passive transfer study in mice against pre-immune group. (XLSX)

S1 Text Example of calculation for K2-Rhavi-FlaBD2-MrkA HMW, high% MAPS complex Mole protein/Mole K2 repeating unit (RU) ratio. The Protein/K2 w/w ratio is 15.2 (HPAEC-PAD/microBCA ratio of the final purified MAPS). The number of moles of protein dimer is: [15.2 mg/(57.25 x 2) kDa] = 0.133 µmole. The number of moles of K2 RU is: (1 mg/662.59 Da) x 1000 = 1.509 µmole. The mole protein/mole K2 RU ratio is: (0.133 µmole of protein/1.509 µmole of K2 RU) = 0.088. This ratio would correspond to 8.8% of K2 RU biotinylated (experimental value from biotin quantitation analysis on the biotinylated PS is 7.9%).
(DOCX)
