# Supplementary figures and images for: Understanding the impact of Klebsiella pneumoniae K-Antigen based MAPS vaccine design on the immune response in animal models
Source: PLoS Pathog. 2026 Jun 10;22(6):e1014289. doi: 10.1371/journal.ppat.1014289 (PMC13252833; doi:10.1371/journal.ppat.1014289)

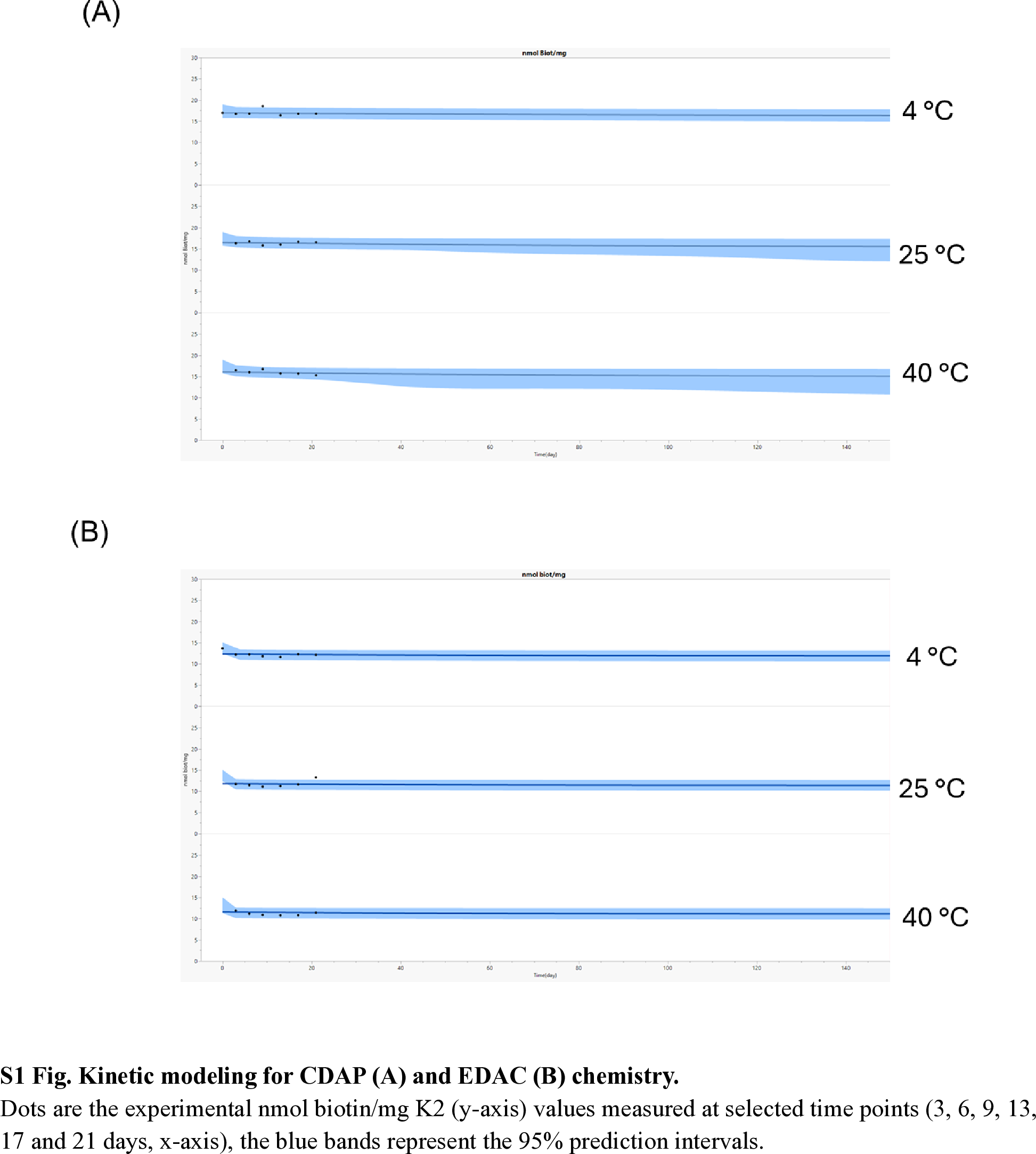

Supplement: S1 Fig — Dots are the experimental nmol biotin/mg K2 (y-axis) values measured at selected time points (3, 6, 9, 13, 17 and 21 days, x-axis). (TIF) [file ppat.1014289.s001.tif]

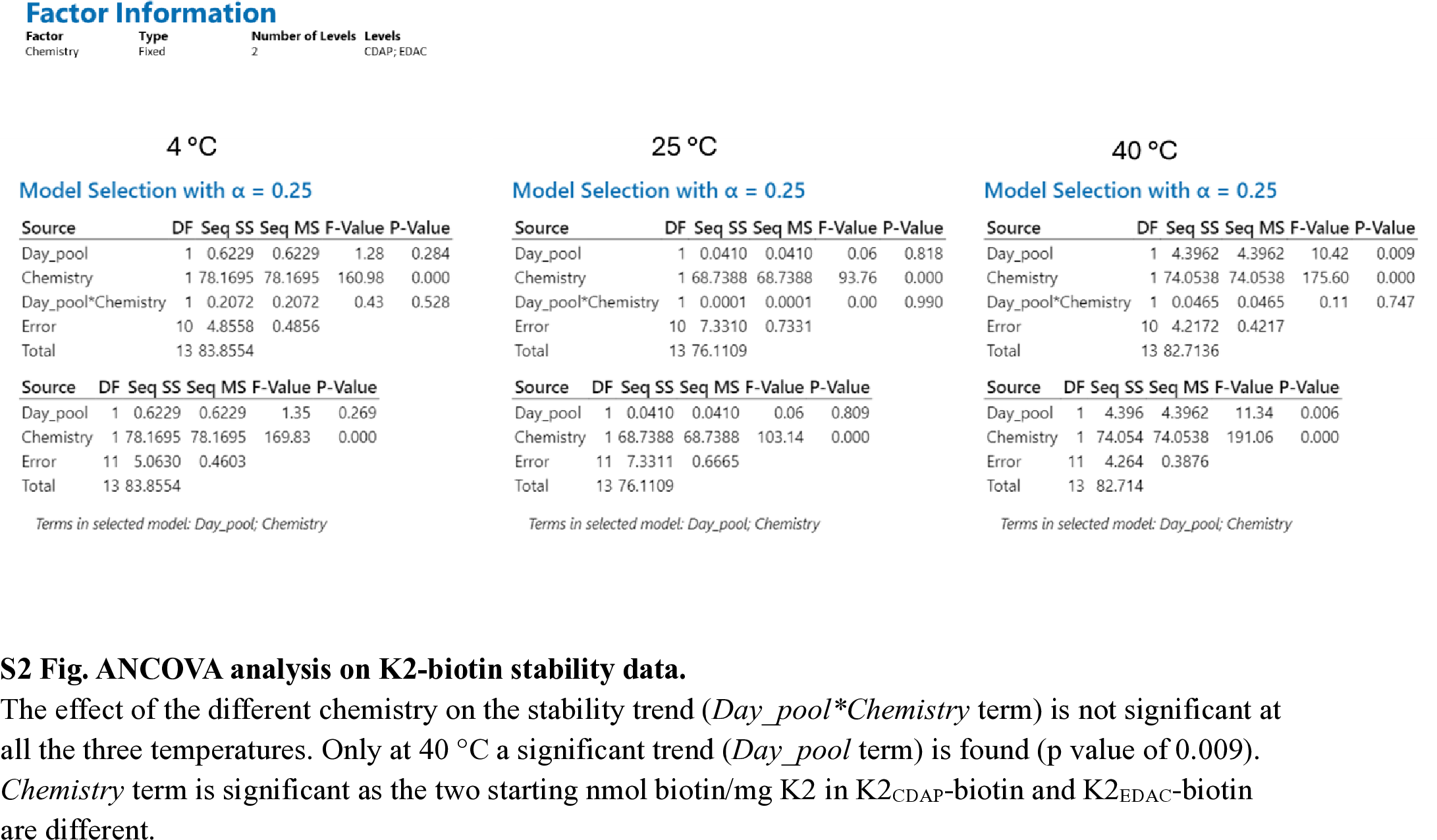

Supplement: S2 Fig — The effect of the different chemistry on the stability trend (Day_pool*Chemistry term) is not significant at all the three temperatures. Only at 40 °C a significant trend (Day_pool term) is found (p value of 0.009). Chemistry term is significant as the two starting nmol biotin/mg K2 in K2CDAP-biotin and K2EDAC-biotin are different. (TIF) [file ppat.1014289.s002.tif]

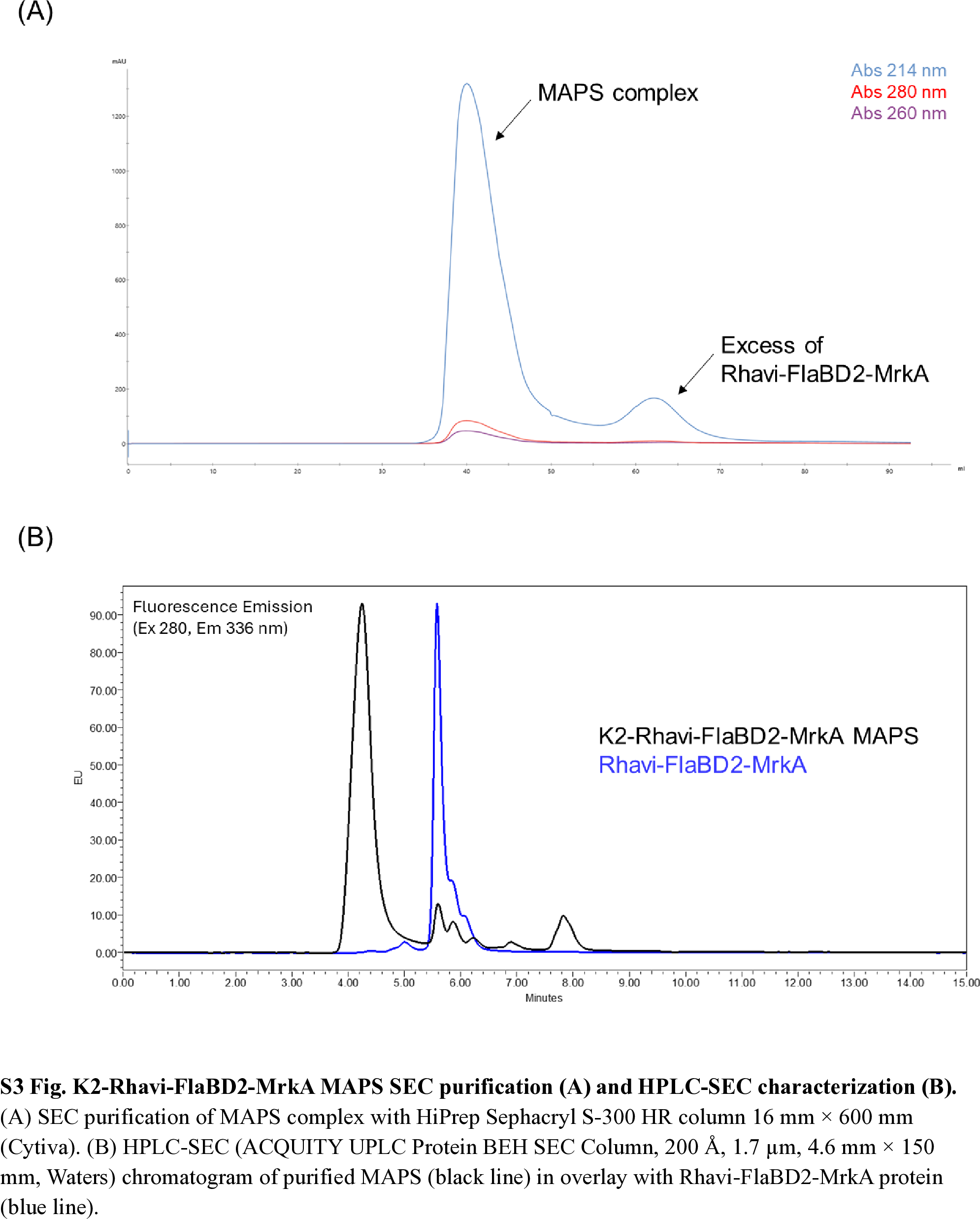

Supplement: S3 Fig — (A) SEC purification of MAPS complex with HiPrep Sephacryl S-300 HR column 16 mm × 600 mm (Cytiva). (B) HPLC-SEC (ACQUITY UPLC Protein BEH SEC Column, 200 Å, 1.7 µm, 4.6 mm × 150 mm, Waters) chromatogram of purified MAPS (black line) in overlay with Rhavi-FlaBD2-MrkA protein (blue line). (TIF) [file ppat.1014289.s003.tif]

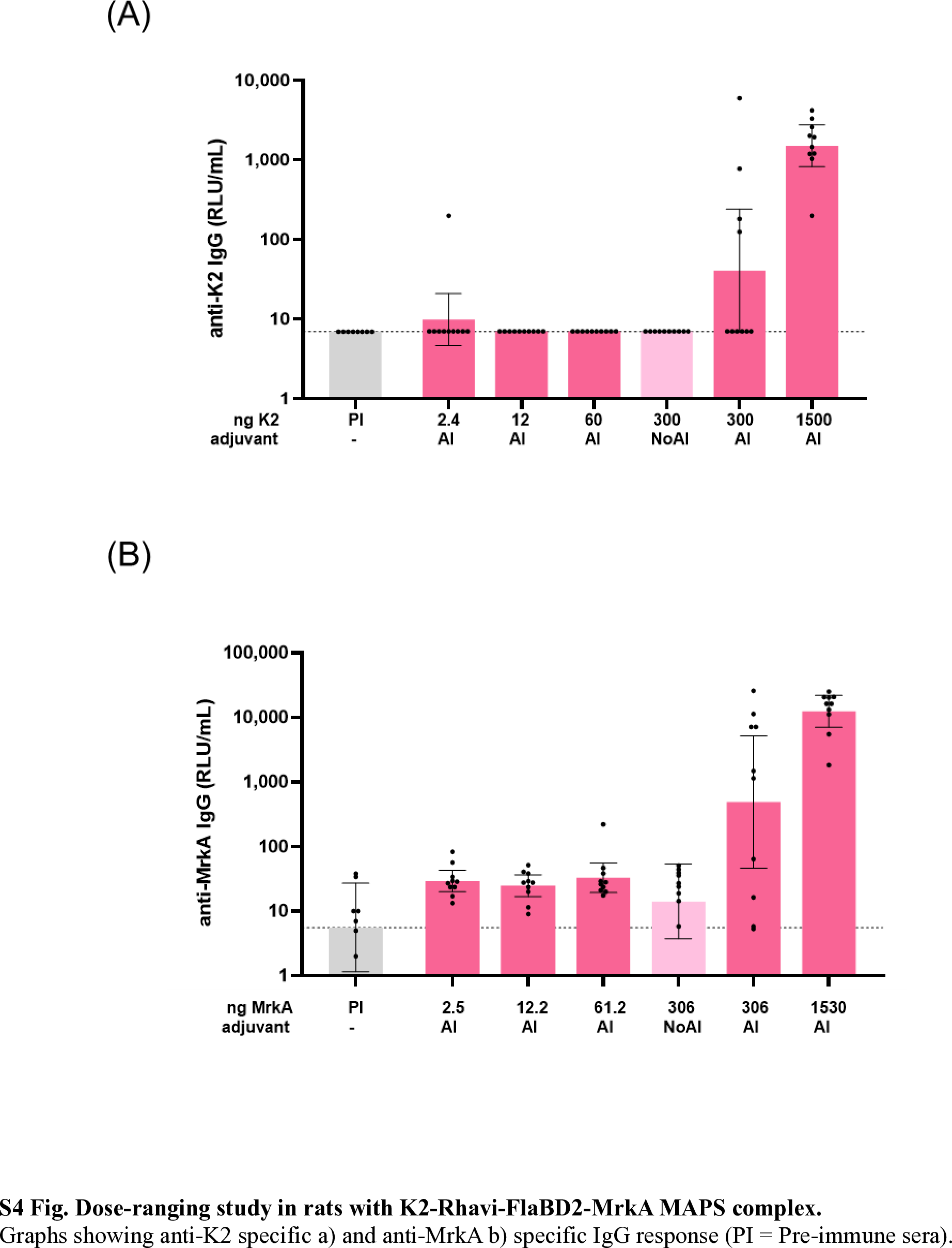

Supplement: S4 Fig — Graphs showing anti-K2 specific (A) and anti-MrkA (B) specific IgG response. (TIF) [file ppat.1014289.s004.tif]

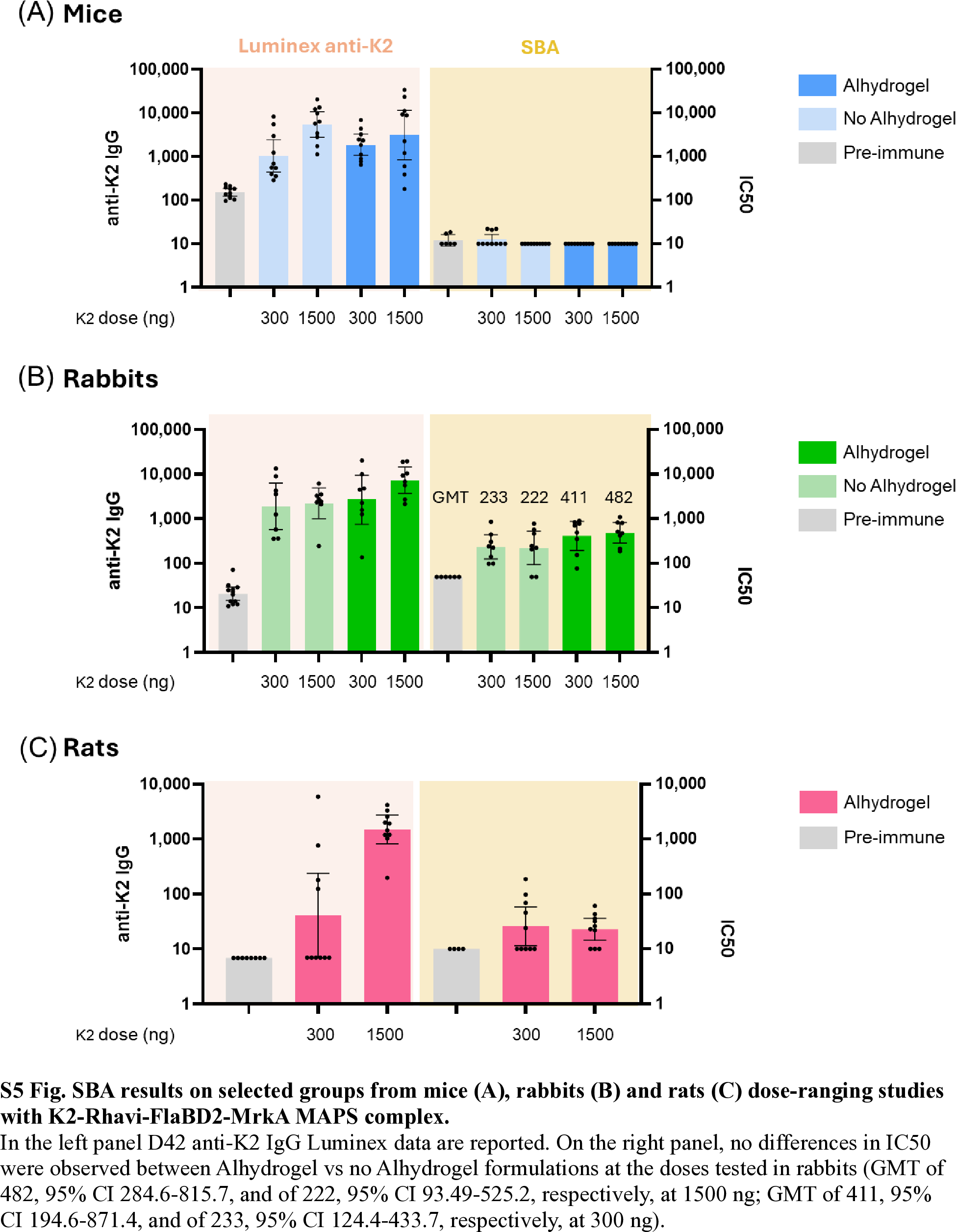

Supplement: S5 Fig — In the left panel D42 anti-K2 IgG Luminex data are reported. No differences were observed between Alhydrogel vs no Alhydrogel formulations at the doses tested in rabbits (GMT of 482, 95% CI 284.6-815.7, and of 222, 95% CI 93.49-525.2, respectively, at 1500 ng; GMT of 411, 95% CI 194.6-871.4, and of 233, 95% CI 124.4-433.7, respectively, at 300 ng). (TIF) [file ppat.1014289.s005.tif]

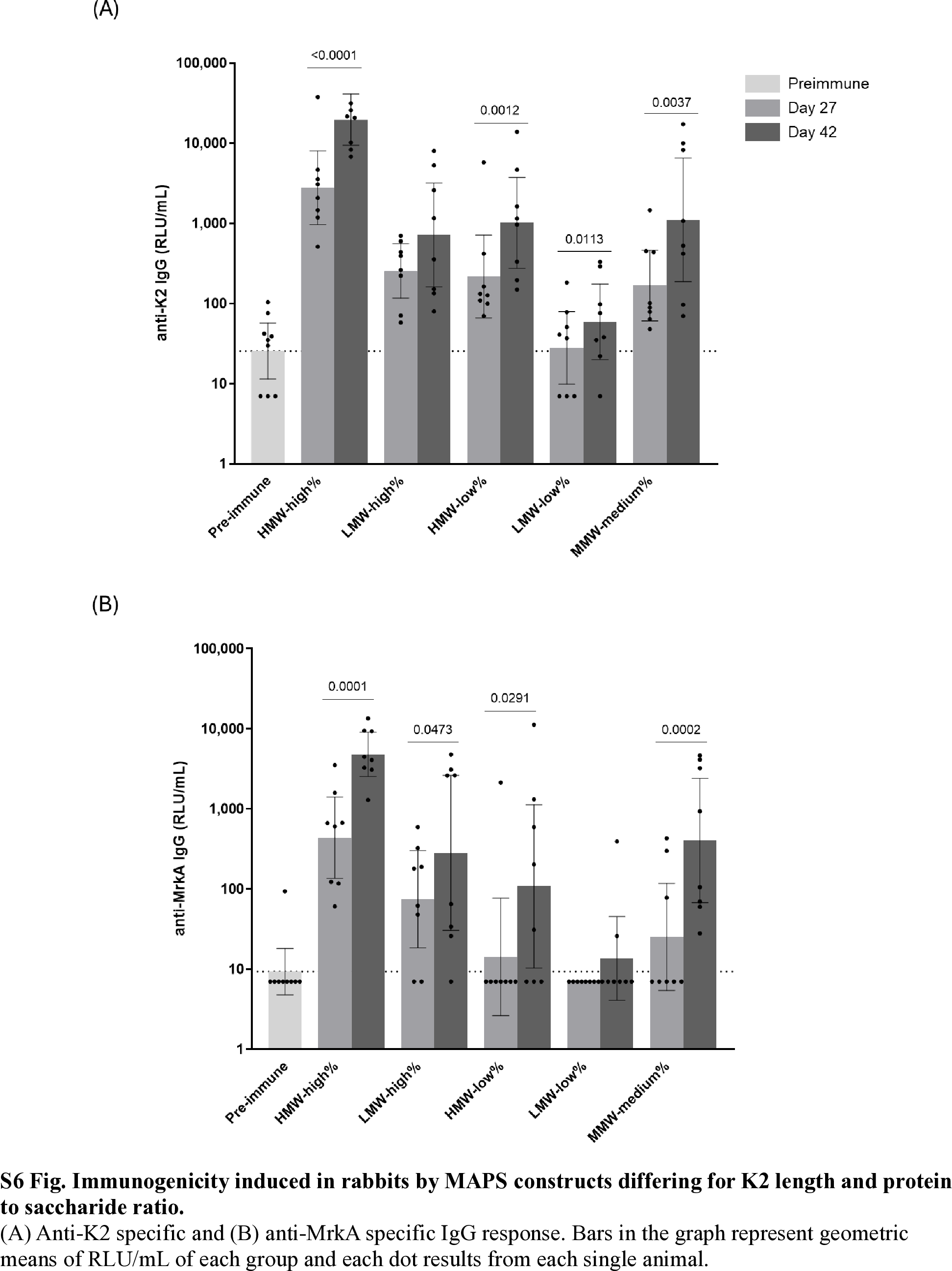

Supplement: S6 Fig — (A) Anti-K2 specific and (B) anti-MrkA specific IgG response. Bars in the graph represent geometric means of RLU/mL of each group and each dot results from each single animal. (TIF) [file ppat.1014289.s006.tif]

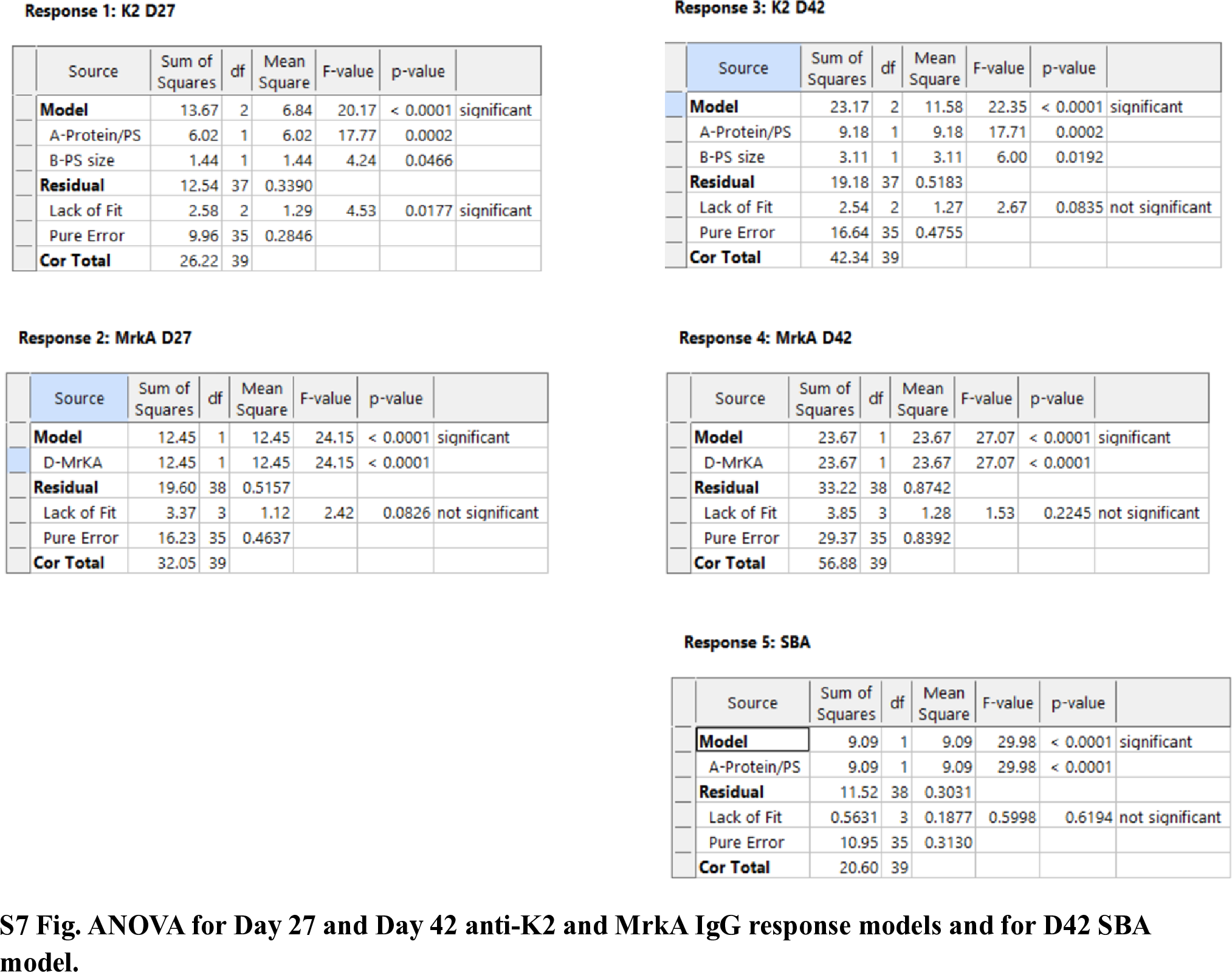

Supplement: S7 Fig — (TIF) [file ppat.1014289.s007.tif]

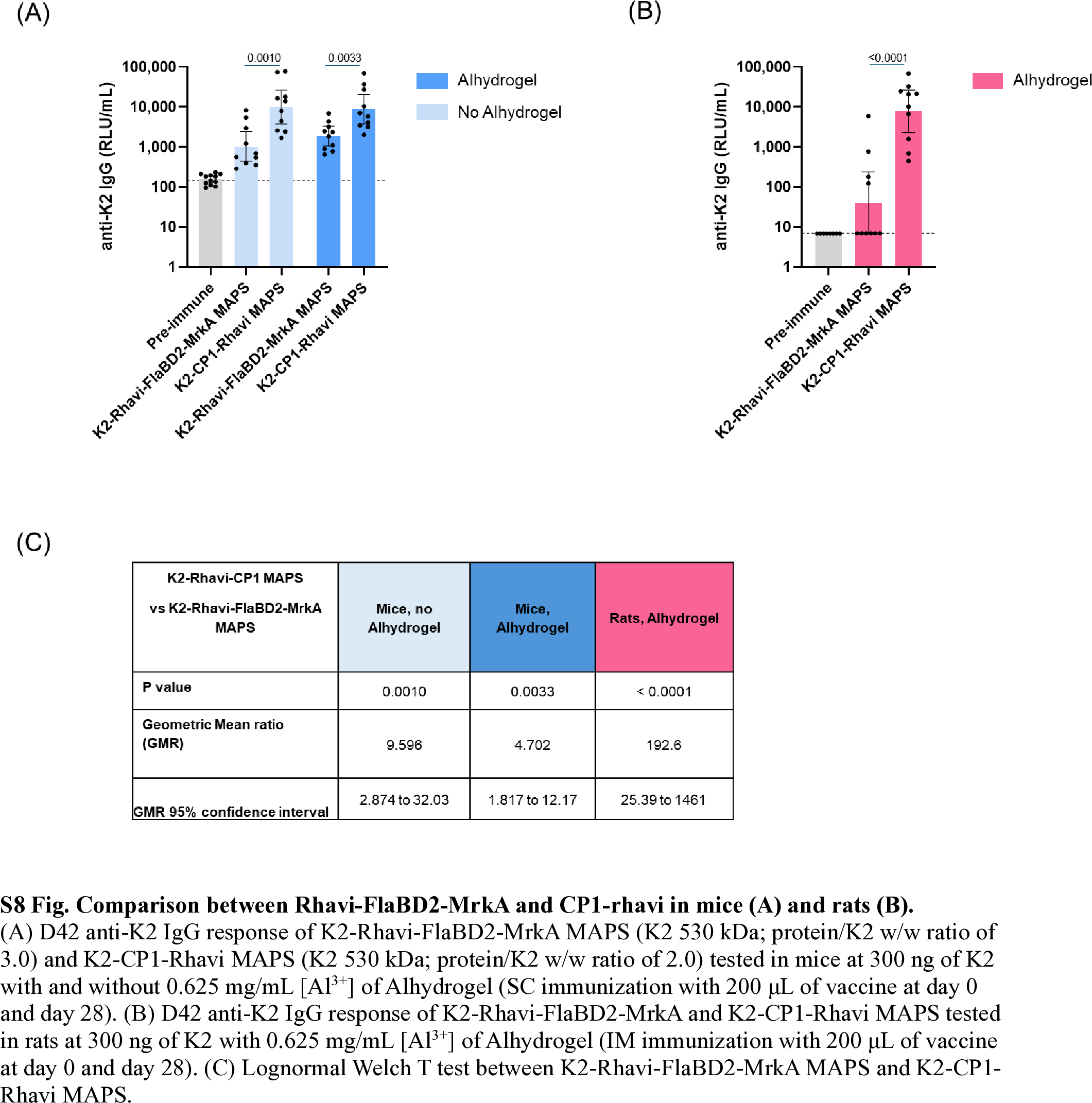

Supplement: S8 Fig — (A) D42 anti-K2 IgG response of K2-Rhavi-FlaBD2-MrkA MAPS (K2 530 kDa; protein/K2 w/w ratio of 3.0) and K2-CP1-Rhavi MAPS (K2 530 kDa; protein/K2 w/w ratio of 2.0) tested in mice at 300 ng of K2 with and without 0.625 mg/mL [Al3+] of Alhydrogel (SC immunization with 200 μL of vaccine at day 0 and day 28). (B) D42 anti-K2 IgG response of K2-Rhavi-FlaBD2-MrkA and K2-CP1-Rhavi MAPS tested in rats at 300 ng of K2 with 0.625 mg/mL [Al3+] of Alhydrogel (IM immunization with 200 μL of vaccine at day 0 and day 28). (C) Lognormal Welch T test between K2-Rhavi-FlaBD2-MrkA MAPS and K2-CP1-Rhavi MAPS. (TIF) [file ppat.1014289.s008.tif]
