# Supplementary material for: Understanding the impact of Klebsiella pneumoniae K-Antigen based MAPS vaccine design on the immune response in animal models
Source: PLoS Pathog. 2026 Jun 10;22(6):e1014289. doi: 10.1371/journal.ppat.1014289 (PMC13252833; doi:10.1371/journal.ppat.1014289)
Supplement: S1 Text — The Protein/K2 w/w ratio is 15.2 (HPAEC-PAD/microBCA ratio of the final purified MAPS). The number of moles of protein dimer is: [15.2 mg/(57.25 x 2) kDa] = 0.133 µmole. The number of moles of K2 RU is: (1 mg/662.59 Da) x 1000 = 1.509 µmole. The mole protein/mole K2 RU ratio is: (0.133 µmole of protein/1.509 µmole of K2 RU) = 0.088. This ratio would correspond to 8.8% of K2 RU biotinylated (experimental value from biotin quantitation analysis on the biotinylated PS is 7.9%). (DOCX) [file ppat.1014289.s015.docx]

**S1 Text. Example of calculation for K2-Rhavi-FlaBD2-MrkA HMW, high% MAPS complex of Mole protein/Mole K2 repeating unit (RU) ratio.**

The Protein/K2 w/w ratio is 15.2 (HPAEC-PAD/microBCA ratio of the final purified MAPS).
The number of moles of protein dimer is: [15.2 mg /(57.25 x 2) kDa] = 0.133 µmole
The number of moles of K2 RU is: (1 mg /662.59 Da) x 1000 = 1.509 µmole
The mole protein/mole K2 RU ratio is: (0.133 µmole of protein /1.509 µmole of K2 RU) = 0.088
This ratio would correspond to 8.8% of K2 RU biotinylated (experimental value from biotin quantitation analysis on the biotinylated PS is 7.9%).
